# Supplementary material for: Cost-effectiveness of Zvandiri, a community-based support intervention to reduce virological failure in adolescents living with HIV in Zimbabwe: Results of a decision analytical model
Source: PLOS Glob Public Health. 2025 Dec 1;5(12):e0005545. doi: 10.1371/journal.pgph.0005545 (PMC12668612; doi:10.1371/journal.pgph.0005545)
Supplement: Attachment — Submitted filename: Response to reviewers request for funding information.docx [file pgph.0005545.s004.docx]

**Response to reviewers comments – Zvandiri CEA**

| **Suggested corrections** |  | **Completion status** |
| --- | --- | --- |
| Dear Dr. Mangenah,  Thank you for submitting your manuscript entitled "Cost-effectiveness of Zvandiri, a community-based support intervention to reduce virological failure in adolescents living with HIV in Zimbabwe: Results of a decision analytical model" to PLOS Global Public Health. Your manuscript files have been checked in-house, but before we can proceed we need you to address the following issues: |  | Done |
| 1. Please confirm at this time whether or not your submission contains all raw data required to replicate the results of your study. Authors must share the “minimal data set” for their submission. PLOS defines the minimal data set to consist of the data required to replicate all study findings reported in the article, as well as related metadata and methods (<https://journals.plos.org/globalpublichealth/s/data-availability#loc-minimal-data-set-definition>).  Authors do not need to submit their entire data set if only a portion of the data was used in the reported study.  If there are ethical or legal restrictions on sharing a de-identified data set, please explain them in detail (e.g., data contain potentially sensitive information, data are owned by a third-party organization, etc.) and who has imposed them (e.g., an ethics committee). | We have uploaded the following spreadsheet file “Zvandiri Markov Model Parameters” to satisfy the minimal dataset required to replicate the modelling study. We have modified the data statement as below | Done |
|  | A spreadsheet (Zvandiri Markov Model Parameters) with all model data pertinent to the cost effectiveness analysis has been submitted together with the manuscript and other supporting materials. Potentially sensitive data for the cohort of individual adolescents living with HIV used to estimate the transition probabilities is held and available upon reasonable request via a non-author point of contact:  Institution: Centre for Sexual Health and HIV/AIDS Research (CeSHHAR)  Contact person: Data Manager, Jeffrey Dirawo (Jeffrey.dirawo@ceshhar.org). | Done |
| 2. Thank you for submitting your Financial Disclosure. Please revise the current statement, ensuring it complies with the following PLOS requirements: | We have revised the current statement to have the below information | Done |
| a) Acronyms should be spelled out | We have checked to ensure and can confirm that there are no acronyms in our financial disclosure statement | Done |
| b) Grant numbers should be included in parentheses after the funder, e.g. “This study was funded by a grant from the National Institutes of Health (NIH-18-93294-739X)” | This (in parentheses) grant number has been inserted (Grant # 1710223/OV192217) | Done |
| c) Website URLs should be included, if necessary | There is no grant website to add | Done |
| d) Use initials to identify authors who received the funding | We have added the initials FMC (Frances Mary Cowan) as the author who received the grant funding | Done |
|  |  |  |
