# Supplementary material for: Cost-effectiveness of Zvandiri, a community-based support intervention to reduce virological failure in adolescents living with HIV in Zimbabwe: Results of a decision analytical model
Source: PLOS Glob Public Health. 2025 Dec 1;5(12):e0005545. doi: 10.1371/journal.pgph.0005545 (PMC12668612; doi:10.1371/journal.pgph.0005545)
Supplement: Attachment — Submitted filename: Response to reviewers_8Nov25.docx [file pgph.0005545.s005.docx]

8^th^ November 2025

Dear Editors

Thank you for the latest suggested corrections that you provided for our manuscript “Cost-

effectiveness of Zvandiri, a community-based support intervention to reduce virological

failure in adolescents living with HIV in Zimbabwe: Results of a decision analytical model” and

for providing us with an opportunity to respond. Please find in the table below, a line-by-line

response to each of the suggested corrections and how we have incorporated them into the

manuscript. In addition to the marked up corrections each response highlights the line

numbers in the clean manuscript version. We would like to draw your attention to the fact

that the system is crushing when we click “proceed” with the old manuscript files and

figures removed. We have therefore dated the revised files 8 November 2025.

Yours sincerely,

Collin Mangenah

| Dear Dr. Collin Mangenah,  Thank you for submitting your manuscript to PLOS Global Public Health. After careful consideration, we feel that it has merit but does not fully meet PLOS Global Public Health’s publication criteria as it currently stands. Therefore, we invite you to submit a revised version of the manuscript that addresses the points raised during the review process.  ==============================  Please submit your revised manuscript by November 11, 2025. If you will need more time than this to complete your revisions, please reply to this message or contact the journal office at [globalpubhealth@plos.org](mailto:globalpubhealth@plos.org). When you're ready to submit your revision, log on to <https://www.editorialmanager.com/pgph/> and select the 'Submissions Needing Revision' folder to locate your manuscript file.  Please include the following items when submitting your revised manuscript:   - A rebuttal letter that responds to each point raised by the editor and reviewer(s). You should upload this letter as a separate file labeled 'Response to Reviewers'. - A marked-up copy of your manuscript that highlights changes made to the original version. You should upload this as a separate file labeled 'Revised Manuscript with Track Changes'. - An unmarked version of your revised paper without tracked changes. You should upload this as a separate file labeled 'Manuscript'.   Guidelines for resubmitting your figure files are available below the reviewer comments at the end of this letter.  We look forward to receiving your revised manuscript.  Kind regards,  Vishal Goyal Academic Editor PLOS Global Public Health |  |  |
| --- | --- | --- |
| **Suggested corrections** |  | **Completion status** |
| Journal Requirements: |  |  |
| 1. Please note that PLOS Global Public Health has specific guidelines on code sharing for submissions in which author-generated code underpins the findings in the manuscript. In these cases, all author-generated code must be made available without restrictions upon publication of the work. Please review our guidelines at <https://journals.plos.org/globalpublichealth/s/materials-and-software-sharing#loc-sharing-code> and ensure that your code is shared in a way that follows best practice and facilitates reproducibility and reuse. | All author-generated code will be made available without restrictions upon publication of the work as per request. | Done |
| 2. We noticed you have some minor occurrence of overlapping text with the following previous publication(s), which needs to be addressed: |  | Done |
| -DOI:10.1016/j.jval.2015.09.2940 | We have edited two sentences in the background which relate to the objective and conclusion of Kessler et als cited work. | Done |
|  | A study in East Africa evaluated the cost-effectiveness of the most effective retention-in-care strategies and to contrast them with alternative resource allocation options for individuals living with HIV, particularly in relation to expansion of access to ART [14] (lines 70-73).  The study findings suggest possibility of scaling up retention programmes at relatively low cost, which would serve as a valuable addition to the options available for policy and decision-makers in the East African region [14]. (Lines 73-75) | Done |
| -DOI:10.1097/qad.0000000000003440 | It is unclear to which this section relates but we have edited the following sections in the background section to avoid similarities with the main trial paper by Mavhu et al | Done |
|  | Scalable, effective and cost-effective strategies for differentiated support maximising not only wellbeing but adherence to treatment and care are needed (Lines 56 to 57) | Done |
|  | Primary care facilities however frequently lack adequate capacity to provide effective support for adherence as well as the broader psychosocial issues that children and adolescents grapple with (Lines 57 to 59) | Done |
|  | Several analyses have explored the effectiveness of community-based support programmes which the World Health Organisation recommends to improve the wellbeing and longevity of ALHIV in Eastern and Southern Africa (Lines 59 to 62) | Done |
|  | Policymaking decisions regarding adoption of public health interventions must however consider not only effectiveness but value for money particularly given the current international HIV funding crisis (Lines 62 to 64) | Done |
| In your revision ensure you cite all your sources (including your own works), and quote or rephrase any duplicated text outside the methods section. Further consideration is dependent on these concerns being addressed. | We have rephrased the below sentences | Done |
|  | As the proportion of adolescents living with HIV (ALHIV, aged 10-19) continues to increase globally, largely because more perinatally infected children are surviving into adolescence, a significant concern arises from their higher attrition rates from HIV treatment and care compared to children and adults, leading to greater instances of treatment failure, morbidity, and mortality (Lines 48 to 52) | Done |
|  | This is especially noticeable in sub-Saharan Africa (SSA), which is home to 84% of the world's ALHIV (still projected to reach 50% by 2050) and where adolescents are the only demographic experiencing rising mortality rates in this region (Lines 52 to 54) | Done |
| 3. If the reviewer comments include a recommendation to cite specific previously published works, please review and evaluate these publications to determine whether they are relevant and should be cited. There is no requirement to cite these works unless the editor has indicated otherwise. | Thank you. This is noted. | Done |
| 4. Please review your reference list to ensure that it is complete and correct. If you have cited papers that have been retracted, please include the rationale for doing so in the manuscript text, or remove these references and replace them with relevant current references. Any changes to the reference list should be mentioned in the rebuttal letter that accompanies your revised manuscript. If you need to cite a retracted article, indicate the article’s retracted status in the References list and also include a citation and full reference for the retraction notice. | Thank you. This is noted. | Done |
| Reviewers' comments: |  |  |
| **Comments to the Author** |  |  |
| Reviewer #1: The authors rebuttal letter states that they attended to the minor comments however, none of the corrections are reflected in the revised manuscript. The authors need to revise the manuscript accordingly. | We have rehighlighted the minor changes made (page numbering refers to the clean version) in the tracked changed version | **Done** |
| **Minor revisions required as highlighted in the attached document.** |  |  |
|  | Affiliation has been double checked and is accurate | Done |
| Would | “Would” replaces “will” in line 39 | Done |
| Reference style? Not [1-6]? Check rest of document | Referencing has been corrected e.g lines 52, 67, 73 and throughout the document | Done |
| Or rather effective in increasing rates of viral suppression? Rephrase for clarity. | Line 100 rephrased to “increasing rates of viral suppression” instead of “reducing rates of viral non-suppression” | Done |
| spacing | Spacing inserted into line 149 | Done |
| Not defined | SoC now fully defined as “standard of care” at first mention in line 107 | Done |
| Were | “were” inserted to replace “was” in line 188 | Done |
| Also referred to as standard of care (SoC)? Consistency is preferred | “Standard care” corrected to “standard of care” line 237 | Done |
| SoC (Standard of Care)? Check rest of the document. | “Standard care” replaced with acronym “SoC” in line 483 as previously defined at first mention above in line 174 | Done |
| One word | Corrected to one word in lines 122 and 562 | Done |
| Table 1 heading talks about three states which are not explained here but only defined later. Instead, 13 states are shown in the table.  Rather introduce Figure 1 before Table 1 for clarity. | In lines 182 to 186 we now introduce Figure 1 and precede it with the following sentence introducing figure one “In the decision-analytic model, to appropriately assign QALYs to all patients who are alive, we modified the primary outcome by separating the proportion of participants who had virological failure from those who had died (and excluding non-HIV related deaths) as they could therefore still transition back to states A (HIV viral load undetected) and B (HIV viral load <1000 copies/mL) (Figure 1)” | Done |
| RCT not defined | RCT written out in full to “randomised control trial” (Line 474) | Done |
| Reviewer #2: The authors made a more clear explanation of the statistical methods and parts of the discussion. This improved the quality of the manuscript. | Thank you | Done |
| [NOTE: If reviewer comments were submitted as an attachment file, they will be attached to this email and accessible via the submission site. Please log into your account, locate the manuscript record, and check for the action link "View Attachments". If this link does not appear, there are no attachment files.] | Noted. There are no attached files and so we have worked with the files in the system | Done |
| Figure Resubmissions: |  | Done |
| While revising your submission, we strongly recommend that you use PLOS’s NAAS tool (<https://ngplosjournals.pagemajik.ai/artanalysis>) to test your figure files. NAAS can convert your figure files to the TIFF file type and meet basic requirements (such as print size, resolution), or provide you with a report on issues that do not meet our requirements and that NAAS cannot fix. | We have now used PLOS’s NAAS tool to generate TIFF figure files | Done |
| After uploading your figures to PLOS’s NAAS tool - <https://ngplosjournals.pagemajik.ai/artanalysis>, NAAS will process the files provided and display the results in the "Uploaded Files" section of the page as the processing is complete. If the uploaded figures meet our requirements (or NAAS is able to fix the files to meet our requirements), the figure will be marked as "fixed" above. If NAAS is unable to fix the files, a red "failed" label will appear above. When NAAS has confirmed that the figure files meet our requirements, please download the file via the download option, and include these NAAS processed figure files when submitting your revised manuscript. | We have now used PLOS’s NAAS tool to generate, download and upload the required TIFF format (Please see above response) | Done |
| **Other changes** |  |  |
|  | Lines 586-588 “The Zvandiri intervention should continue to be scaled up nationally and regionally (now adopted / localised in 14 countries).” | Done |
| 1. Please amend your detailed Financial Disclosure statement. This is published with the article. It must therefore be completed in full sentences and contain the exact wording you wish to be published. | Our financial disclosure statement is fully written out (Please see below response) | Done |
| 1. Please clarify all sources of financial support for your study. List the grants, grant numbers, and organizations that funded your study, including funding received from your institution. Please note that suppliers of material support, including research materials, should be recognized in the Acknowledgements section rather than in the Financial Disclosure. | The following statement outlines the source of financial support for our costing study. “The research reported in this publication was funded by ViiV Healthcare's Positive Action for Adolescents Programme (Grant # 1710223/OV192217 to FMC). Funders played no role in study design, data collection and analysis, the decision to publish, or preparation of the manuscript. The content is solely the responsibility of the authors and does not necessarily represent the official views of ViiV Healthcare.” | Done |
| 2. State the initials, alongside each funding source, of each author to receive each grant. For example: "This work was supported by the National Institutes of Health (####### to AM; ###### to CJ) and the National Science Foundation (###### to AM)." | The initials of FMC are given as the recipient of study funding as shown in the above response | Done |
| 3. State what role the funders took in the study. If the funders had no role in your study, please state: “The funders had no role in study design, data collection and analysis, decision to publish, or preparation of the manuscript.” | We have now indicated that “The funders had no role in study design, data collection and analysis, decision to publish, or preparation of the manuscript.” Please see above response | Done |
| 4. If any authors received a salary from any of your funders, please state which authors and which funders.  If you did not receive any funding for this study, please simply state: “The authors received no specific funding for this work.” | We have now indicated that “The authors received no specific funding for this work.” | Done |
| 2. Please remove the old manuscript file and retain only the revised files. | The system is crushing when we click “proceed” with the old manuscript files and figures removed. We have therefore dated the revised files 8 November 2025. |  |
| 3. We have noticed that you have uploaded Supporting Information files, but you have not included a list of legends. Please add a full list of legends for your Supporting Information files before or after the references list. | We have added a full list of figures before the references list. |  |
